# Hepatic deletion of p110α and p85α results in insulin resistance despite sustained IRS1-associated phosphatidylinositol kinase activity

**DOI:** 10.12688/f1000research.12418.2

**Published:** 2018-06-05

**Authors:** Aditi Chaudhari, Katarina Ejeskär, Yvonne Wettergren, C. Ronald Kahn, Victoria Rotter Sopasakis

**Affiliations:** 1 Wallenberg Laboratory, Department of Molecular and Clinical Medicine, University of Gothenburg, Gothenburg, Sweden; 2 Department of Clinical Chemistry and Transfusion Medicine, University of Gothenburg, Gothenburg, Sweden; 3 Institute of Health and Education, Translational Medicine, University of Skövde, Skövde, Sweden; 4 Department of Surgery, University of Gothenburg, Sahlgrenska University Hospital/Östra, Gothenburg, Sweden; 5 Joslin Diabetes Center and Harvard Medical School, One Joslin Place, Boston, MA, USA

**Keywords:** phosphatidylinositol-4, 5-bisphosphate 3-kinase, p110, p85, insulin receptor substrate, insulin resistance, glucose intolerance

## Abstract

**Background**: Class IA phosphatidylinositol-4,5-bisphosphate 3-kinase (PI3K) is an integral mediator of insulin signaling. The p110 catalytic and p85 regulatory subunits of PI3K are the products of separate genes, and while they come together to make the active heterodimer, they have opposing roles in insulin signaling and action. Deletion of hepatic p110α results in an impaired insulin signal and severe insulin resistance, whereas deletion of hepatic p85α results in improved insulin sensitivity due to sustained levels of phosphatidylinositol (3,4,5)-trisphosphate. Here, we created mice with combined hepatic deletion of p110α and p85α (L-DKO) to study the impact on insulin signaling and whole body glucose homeostasis.

**Methods**: Six-week old male flox control and L-DKO mice were studied over a period of 18 weeks, during which weight and glucose levels were monitored, and glucose tolerance tests, insulin tolerance test and pyruvate tolerance test were performed. Fasting insulin, insulin signaling mediators, PI3K activity and insulin receptor substrate (IRS)1-associated phosphatidylinositol kinase activity were examined at 10 weeks. Liver, muscle and white adipose tissue weight was recorded at 10 weeks and 25 weeks.

**Results**: The L-DKO mice showed a blunted insulin signal downstream of PI3K, developed markedly impaired glucose tolerance, hyperinsulinemia and had decreased liver and adipose tissue weights. Surprisingly, however, these mice displayed normal hepatic glucose production, normal insulin tolerance, and intact IRS1-associated phosphatidylinositol kinase activity without compensatory upregulated signaling of other classes of PI3K.

**Conclusions**: The data demonstrate an unexpectedly overall mild metabolic phenotype of the L-DKO mice, suggesting that lipid kinases other than PI3Ks might partially compensate for the loss of p110α/p85α by signaling through other nodes than Akt/Protein Kinase B.

## Introduction

Class IA phosphatidylinositol-4,5-bisphosphate 3-kinase (PI3K) is a central mediator of a number of membrane receptor signaling pathways, including the insulin signaling pathway ^1, 2^. Following receptor activation by insulin, PI3K binds to tyrosine-phosphorylated amino acids of the insulin receptor substrates (IRS), resulting in PI3K activation and the formation of phosphatidylinositol (3,4,5)-trisphosphate (PIP _3_). PIP _3_ has high affinity for the pleckstrin homology (PH) domain of the downstream target Akt/Protein Kinase B (PKB). The interaction of PIP _3_ with the PH domain enables phosphorylation of Akt/PKB by phosphoinositide dependent kinase (PDK) 1 and PDK 2, ultimately triggering a number of metabolic actions, such as lipogenesis, glycogen synthesis, inhibition of hepatic glucose output and increased glucose uptake in muscle and adipose tissue.

Class IA PI3Ks consist of two subunits. The catalytic subunit, p110, contains the kinase domain responsible for the formation of PIP _3_. The regulatory subunit, the most common of which is p85α, binds to phosphorylated tyrosine residues in tyrosine kinases and their substrate proteins via its SH2 domain, leading to activation of PI3K activity. Both the regulatory and catalytic subunits exist as several different isoforms. In humans, there are four known catalytic subunit isoforms: p110α, p110β, p110δ and p37δ. p110α, p110β and p110δ are encoded by three different genes, *PIK3CA*, *PIK3CB* and *PIK3CD*, respectively (reviewed in 1), whereas p37δ ( *PIK3CD*_ *v*2) is a splice variant of p110δ ^3, 4^. We and others have shown that of these catalytic subunits, p110α is the major contributor for transmitting the insulin signal ^5– 7^, whereas p110β becomes active primarily in response to G protein-coupled receptor signaling and plays a role in proliferation ^8, 9^. p110δ is more cell specific than p110α and p110β, and plays an important role in immune cells and the embryonic nervous system ^10– 12^.

The regulatory subunits are also encoded by three genes, *PIK3R1*, *PIK3R2* and *PIK3R3*. Their primary gene products are p85α, p85β and p55γ, respectively (reviewed in 1). *PIK3R1* also encodes two splice variants of p85α, p55α and p50α, which have more limited tissue distribution. p85α is the major regulatory subunit isoform, constituting 65%–75% of the intracellular pool of regulatory subunits in most cells ^13^. Despite the crucial role of the regulatory subunits of Class IA PI3K in mediating insulin-dependent PI3K signaling ^14, 15^, mice with a knockout (KO) of the p85α regulatory subunit display increased insulin sensitivity, increased levels of PIP _3_ lipids, elevated Akt/PKB activity and improved glucose tolerance ^13, 16– 19^. The molecular mechanisms that underlie this negative regulation by p85 appear to be complex and include unbalanced stoichiometry between subunits ^20, 21^; effects of p85 on both protecting p110 from degradation while partially inhibiting its kinase activity ^16, 19, 21, 22^; retention of PI3K in an inactive vesicle compartment ^23^; links between p85α and PTEN activity ^24^; links between p85α and JNK activity leading to IRS1 serine phosphorylation and inhibition of IRS1-mediated effects, and links between p85α and XBP-1 in modifying the unfolded protein response ^25^.

To dissect the intricate equilibrium between the catalytic and regulatory subunits of PI3K, as well as the opposing and complex roles of p110α and p85α in insulin signaling and action, in the present study, we have investigated the impact of a combined hepatic deletion of p110α and p85α on insulin signaling and whole body glucose homeostasis.

## Methods

### Animals

All mice in this study were on a 129Sv-C57Bl/6 mixed genetic background. To create the liver double knock-out mice, p110α lox-lox mice ^7^ were crossed with p85α lox-lox mice, hemizygous for the Albumin-Cre recombinase transgene ^14^. Mice were housed on a 12-hour light cycle and fed a standard rodent chow and water *ad libitum*. All protocols for animal use and euthanasia were approved by the Gothenburg Ethical Committee on Animal Experiments, in accordance with Swedish guidelines and Directive 2010/63/EU for animal experiments, and by the Animal Care Use Committee of the Joslin Diabetes Center and Harvard Medical School in accordance with National Institutes of Health guidelines. All efforts were made to ameliorate any suffering of the mice by reducing stress, hosting mice in small groups with items that stimulate their natural activity, and allowing the mice to recover 1–2 weeks after each procedure (such as measuring blood glucose, glucose tolerance test etc). During the insulin tolerance test, the mice were monitored closely to not fall too low in blood glucose levels.

For each experiment, a group of 5–12 male mice per genotype were used. The mice were studied from 6 weeks of age to 25 weeks of age. During this time weight and fasting blood glucose levels were measured every two weeks. Glucose tolerance test was performed at 8 weeks, 16 weeks and 24 weeks. Pyruvate tolerance test was performed at 15 weeks and insulin tolerance test was performed at 19 weeks.

### Metabolic and physiological procedures

Animals were fasted overnight and anesthetized with 2-2-2 tribromoethanol (Sigma-Aldrich, St Louis MO), followed by injection of 5 U of insulin (Actrapid, Novo Nordisk Inc., Plainsboro Township, NJ) or saline via the inferior vena cava. Five minutes after the injection, the liver, muscle and white adipose tissue (WAT) were excised, weighed, and snap-frozen in liquid nitrogen.

Glucose tolerance test was performed by intraperitoneal (i.p.) injection of 2 g glucose/kg BW after an overnight fast. Insulin tolerance test was performed by i.p. injection of 1.25 U insulin/kg BW. Pyruvate tolerance test was performed by i.p. injection of 2 g of pyruvate/kg BW after an over-night fast. Insulin and glucagon was measured with ELISA (Crystal Chem Inc., Downer Grover, IL).

### RNA extraction and gene expression analysis

RNA was extracted by homogenization of liver tissue in RLT buffer (Qiagen, Valencia, CA) followed by extraction using the RNeasy kit (Qiagen, Valencia, CA). For gene analysis, cDNA was prepared using a high capacity cDNA archive kit (Applied Biosystems, Foster City, CA) with random hexamer primers. Gene expression was analyzed by real-time reverse transcription-PCR (RT-PCR) on an ABI Prism sequence detection system (Applied Biosystems, Foster City, CA). The cycling conditions used were an initial 95°C 10-minute step followed by 40 cycles of 95°C for 15s and 60°C for 60s. Samples were normalized to the 18S rRNA gene. Primer sequences are available in Table 1.

**Table 1.** Primer used for gene expression analysis.

Gene	Type	Sequence/Assay ID#	
*Pik3ca*	Gene expression assay	Mm00435673_m1	Thermo Fisher Scientific
*Pik3cb*	Gene expression assay	Mm00659576_m1	Thermo Fisher Scientific
*Pik3r1*	Gene expression assay	Mm01282781_m1	Thermo Fisher Scientific
*Pik3r2*	Gene expression assay	Mm00435694_m1	Thermo Fisher Scientific
*G6pc*	Forward Primer	5’-ACTGTGGGCATCAATCTCCT	
*G6pc*	Reverse Primer	5’-ACAGGTGACAGGGAACTGCT	
*G6pc*	Probe	5’-[6FAM]TGGGTGGCAGTGGTCGG	
*Pck1*	Forward Primer	5’-TGGATGTCGGAAGAGGACTT	
*Pck1*	Reverse Primer	5’-AGTGGCCCCATGCTGAAT	
*Pck1*	Probe	5’-[6FAM]GTGCATGAAAGGCCGCA	
*Fbp1*	Forward Primer	5’-AGCTGCTGAATTCCGCTCTG	
*Fbp1*	Reverse Primer	5’-TTGATCACCAGGTCATTGGA	
*Fbp1*	Probe	5’-[6FAM]GCGGGCATCGCACAGCT	
*18S*	Gene expression assay	4310893E	Thermo Fisher Scientific

### Protein extraction and analysis

Liver tissue was homogenized in lysis buffer containing 25 mM Tris-HCl, 2 mM Na _3_VO4, 10mM Na _4_P _2_O _7_, 1 mM EGTA, 1 mM EDTA, 1% NP-40 and protease inhibitors (Sigma-Aldrich, St Louis MO), then allowed to incubate at 4°C for one hour. Extracts were centrifuged at 55,000 rpm (Beckman 70.1 Ti rotor) for one hour, and the supernatant was stored at -80°C. WAT was homogenized in lysis buffer containing 25 mM Tris-HCl, pH 7.4, 0.5 mM EDTA, 25 mM NaCl, 1% Nonidet P-40, 10 mM NaF, 1 mM orthovanadate, and protease inhibitors (Sigma-Aldrich, St Louis MO) followed by incubation for 2 h at 4°C. The samples were then centrifuged at 12,000 rpm for 15 min, and the supernatant was collected and stored at -80°C. Protein analysis was made by SDS-PAGE and subsequent western blot. Briefly, protein samples were loaded onto 4–12% Bis-Tris protein gels (Thermo-Fisher Scientific, Waltham, MA) and subjected to gel electrophoresis using 25mM Tris, 192 mM glycine and 0.1% SDS as running buffer. Samples were transferred onto a nitrocellulose membrane and the membranes were incubated in 5% skim milk solution for 1 h followed by primary antibody incubation according to the manufacturer’s protocol for each antibody. Membranes were washed 2×5 min and 1×15 min in PBS with 0.1% Tween and then incubated with the secondary antibody for 1h followed by another washing procedure. Immunoprecipitation was performed using magnetic beads coated with protein G (Pierce Biotechnology Inc, Rockford, IL). All western blots and immunoprecipitation experiments were performed with a minimum of four replicates (four separate samples).

### Antibodies

IRS1 (RRID:AB_2127860, rabbit monoclonal, 1:50, Cell Signaling Technology Inc, cat# 3407 for western blot), IRS1 (RRID:AB_631842, rabbit polyclonal, 10μl per reaction, Santa Cruz Biotechnology Inc, cat# sc-559 for immunoprecipitation), p110α (rabbit monoclonal, 1:1000 for western blot and 1:50 for immunoprecipitation, Cell Signaling Technology Inc, cat# 4249), p110β (RRID:AB_2165246, rabbit monoclonal, 1:500 for western blot and 1:50 for immunoprecipitation, Cell Signaling Technology Inc, cat# 3011), p110γ (RRID:AB_10828316, rabbit monoclonal, 1:1000, Cell Signaling Technology Inc, cat# 5405), p110δ (mouse monoclonal, 1:500, Becton, Dickinson and Company, cat# 611015), p101 (RRID:AB_10829448, rabbit monoclonal, 1:1000, Cell Signaling Technology Inc, cat# 5569), Vps34 (RRID:AB_2299765, rabbit monoclonal, 1:1000, Cell Signaling Technology Inc, cat# 4263), p150 (rabbit polyclonal, 1:1000, Cell Signaling Technology Inc, cat# 14580), Akt/PKB (RRID:AB_329827, rabbit polyclonal, 1:1000, Cell Signaling Technology Inc, cat# 9272), pS-Akt/PKB (RRID:AB_329825, rabbit polyclonal, 1:1000, Cell Signaling Technology Inc, cat# 9271), pT-Akt/PKB (RRID:AB_2255933, rabbit monoclonal, 1:1000, Cell Signaling Technology Inc, cat# 2965), pT-p70S6K (RRID:AB_330944, rabbit polyclonal, 1:1000, Cell Signaling Technology Inc, cat# 9205), p70S6K (rabbit polyclonal, 1:500, Cell Signaling Technology Inc, cat# 9202), p85α (RRID:AB_2268174, rabbit monoclonal, 1:1000, Abcam, cat# 22653), p85-pan (RRID:AB_10831521, rabbit monoclonal, 1:1000, Cell Signaling Technology Inc, cat# 4257), pT202/Y204-ERK (RRID:AB_2315112, rabbit monoclonal, 1:1000, Cell Signaling Technology Inc, cat# 4370), ERK (RRID:AB_390779, rabbit monoclonal, 1:1000, Cell Signaling Technology Inc, cat# 4695), p55γ (mouse monoclonal, 1:2000, Abcam, cat# ab186612), PIK3C2α (rabbit polyclonal, 1:1000, MyBiosource, cat# MBS9202698), PIK3C2γ (rabbit polyclonal, 1:1000, MyBiosource, cat# MBS820611). Rabbit secondary antibody (RRID:AB_772206, HRP-linked from donkey, 1:1000, GE Healthcare Life Sciences, cat# NA934), mouse secondary antibody (RRID:AB_772210, HRP-linked from sheep, 1:1000, GE Healthcare Life Sciences, cat# NA931).

### *In vitro* kinase assays

Immunoprecipitates, using protein G Dynabeads (Life Technologies), of liver protein lysates were prepared with p110α and p110β antibodies or IRS1 antibody. The PI3K assay was performed as previously described ^13^. Briefly, immunoprecipitates were incubated with 5 μg PI substrate (phosphatidylinositol from bovine liver), 20 mM MgCl _2_, 8 μM cold ATP and 0.5 μl radio-labeled [γ- ^32^P]-ATP (1.11*10 ^14^ bq/mmol) in PI3K reaction buffer (20 mM Tris-HCl, 100 mM NaCl and 0.5 mM EGTA) for 25 min at room temperature. The resulting radioactively labeled PIP was analyzed with thin layer chromatography and phosphorimaging (FLA-3000, Fujifilm). Prior to these experiments, titration experiments of bead concentration and PI substrate concentration were performed to ensure precipitation of equal amounts of protein, as well as optimal PI concentration to obtain maximal enzyme activity.

### Statistics

All data are presented as mean ± standard error of the mean (SEM). Student's t-test was used for statistical analysis between two unpaired groups. A p-value of <0.05 was considered statistically significant. The statistical software used was GraphPad Prism 7.00.

## Results

### Deletion of hepatic p110α and p85α results in impaired insulin signaling downstream of PI3K

**Figure 1.**
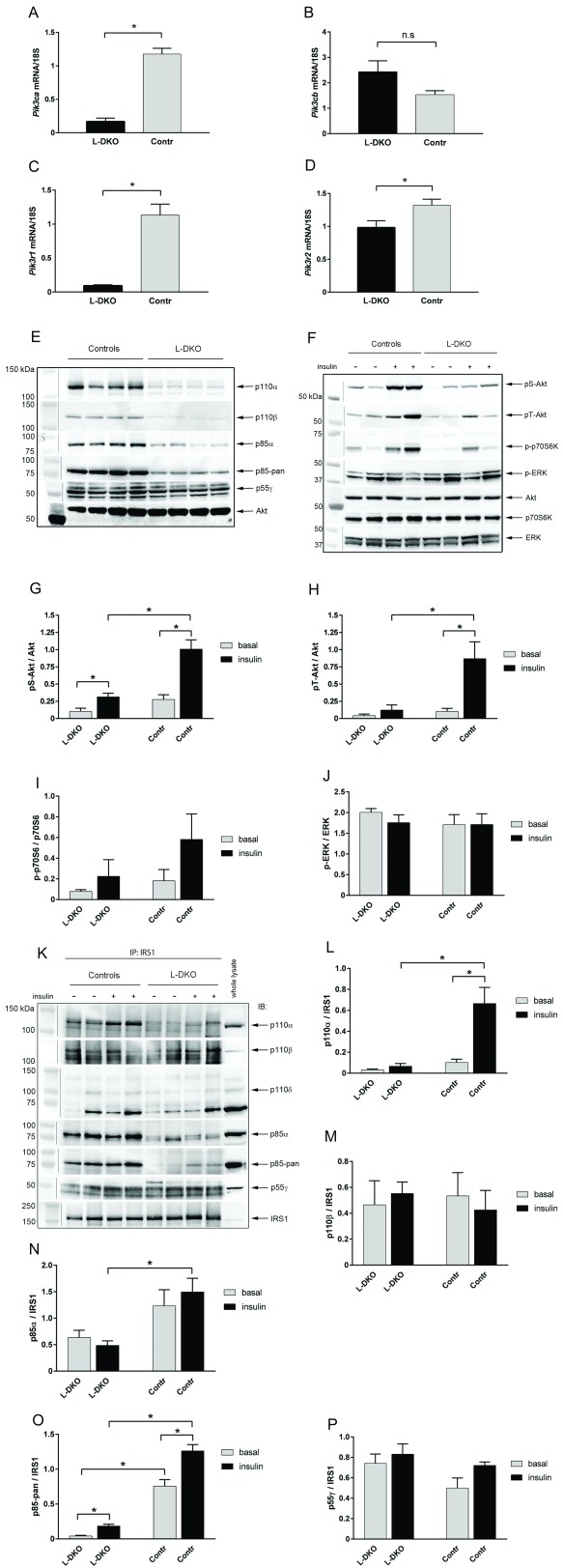
Gene- and protein-expression of regulatory and catalytic subunits of PI3K and associated insulin signaling mediators. mRNA expression of ( **A**) *Pik3ca*, ( **B**) *Pik3cb*, ( **C**) *Pik3r1* and ( **D**) *Pik3r2* in livers of flox controls and L-DKO mice. ( **E**) Representative western blot of protein expression of p110α, p110β, p85α, p85-pan (detects both p85 isoforms) and p55γ. Total Akt was used as a loading control. ( **F**, phosphorylated Akt/PKB (Ser 473 and Thr 308), phosphorylated p70S6 kinase, and phosphorylated ERK in livers of flox controls and L-DKO mice. Total Akt, p70S6K and ERK was used as loading controls. ( **G**– **J**) Quantification measurements of the western blots shown in ( **F**) of pS-Akt, pT-Akt, p-p70S6K and p-ERK respectively. ( **K**) Representative western blot of immunoprecipitation experiments with antibodies for IRS1 and subsequent immunoblotting with antibodies for p110α, p110β, p110δ, p85α, p85-pan (detects both p85 isoforms) and p55γ. The whole-lysate reference sample was from an insulin-treated flox control mouse. ( **L**– **P**) Quantification measurements of the western blots shown in ( **K**) of p110α, p110β, p85α, p85-pan and p55γ. IP = immunoprecipitation, IB = immunoblot. Basal condition is indicated with a minus (-) sign, insulin-treated condition is indicated with a plus (+) sign. Basal conditions refer to fasting of mice overnight and injection of saline through the vena cava. Insulin treatment refers to injection of 5 U of insulin through the vena cava 5 min prior to euthanization. Error bars indicate SEM (n = 5–8). *, p < 0.05 compared to controls. n.s = non significant. Images containing a vertical line are composites taken from a single original image.

Mice with a liver-specific deletion of p110α and p85α, termed hereafter liver double knockout (L-DKO) mice, were created by breeding mice carrying homozygous floxed *Pik3ca* and *Pik3r1* alleles ^14, 26^ with transgenic mice carrying the Cre recombinase driven by the albumin promoter (albumin-Cre). Deletion of *Pik3ca* and *Pik3r1* in the liver resulted in markedly reduced gene and protein expression of p110α and p85α ( Figures 1A, 1C, 1E), as well as impaired activation of the downstream targets Akt/PKB, with decreased phosphorylation of serine 473 and threonine 308, and p70S6 kinase ( Figure 1F–I). p110β gene expression was not affected by the deletion of p110α and p85α ( Figure 1B). p85β gene expression was slightly, but significantly, decreased in the L-DKO livers ( Figure 1D). As expected, in the floxed control mice, there was an increase in the amount of p110α associated with IRS1 in response to insulin compared to basal conditions, whereas no p110α was associated with IRS1 in the L-DKO mice ( Figure 1K and 1L). MAPK signaling, as shown by ERK phosphorylation, was unchanged in the L-DKO mice compared to controls ( Figure 1F and 1J).

Previous studies have shown that the interaction between the regulatory and catalytic subunits of PI3K to form dimers has a mutual stabilizing effect on both subunits, whereas the monomeric forms are more readily subjected to degradation ^19, 21, 22^. We hypothesized that more p85β would bind to IRS1 when p85α was absent, thereby maintaining p110β stabilization. However, only very low levels of p85β protein were detected in the liver of the L-DKO mice, as shown both in assessment of total p85 protein ( Figure 1E) and in p85 immunoprecipitates with IRS1 ( Figure 1K and 1O), similar to what we have reported earlier in liver-specific p85α knock-out mice ^14^. The expression of p55γ regulatory isoform protein was not affected by deletion of p110α and p85α ( Figure 1E), nor was the amount bound to IRS1 ( Figure 1K and 1P). In contrast, total p110β protein expression was decreased in the L-DKO mice compared to controls ( Figure 1E), likely due to destabilization of this catalytic isoform in the absence of p85α, supporting an insufficiency for p85β to compensate for the loss of p85α. Interestingly, despite overall decreased protein expression of p110β, similar amounts of p110β were associated with IRS1 in controls and L-DKO mice ( Figure 1K and 1M). The third catalytic isoform of class IA PI3Ks, p110δ, has been shown to have a major role in immune cells and the embryonic nervous system, but not in other tissues ^10– 12^. Consistent with this, we found only very small amounts of full length p110δ in whole liver lysates or bound to IRS1 ( Figure 1K). However, the antibody picked up a band of about 70 kDa in whole lysates and in IRS1 immunoprecipitates ( Figure 1K). The amount of this protein did not appear to be consistently different between controls and L-DKO mice or between basal and insulin-stimulated samples, so is likely non-specific.

### IRS1-associated phosphatidylinositol kinase activity is intact in the L-DKO mice

**Figure 2.**
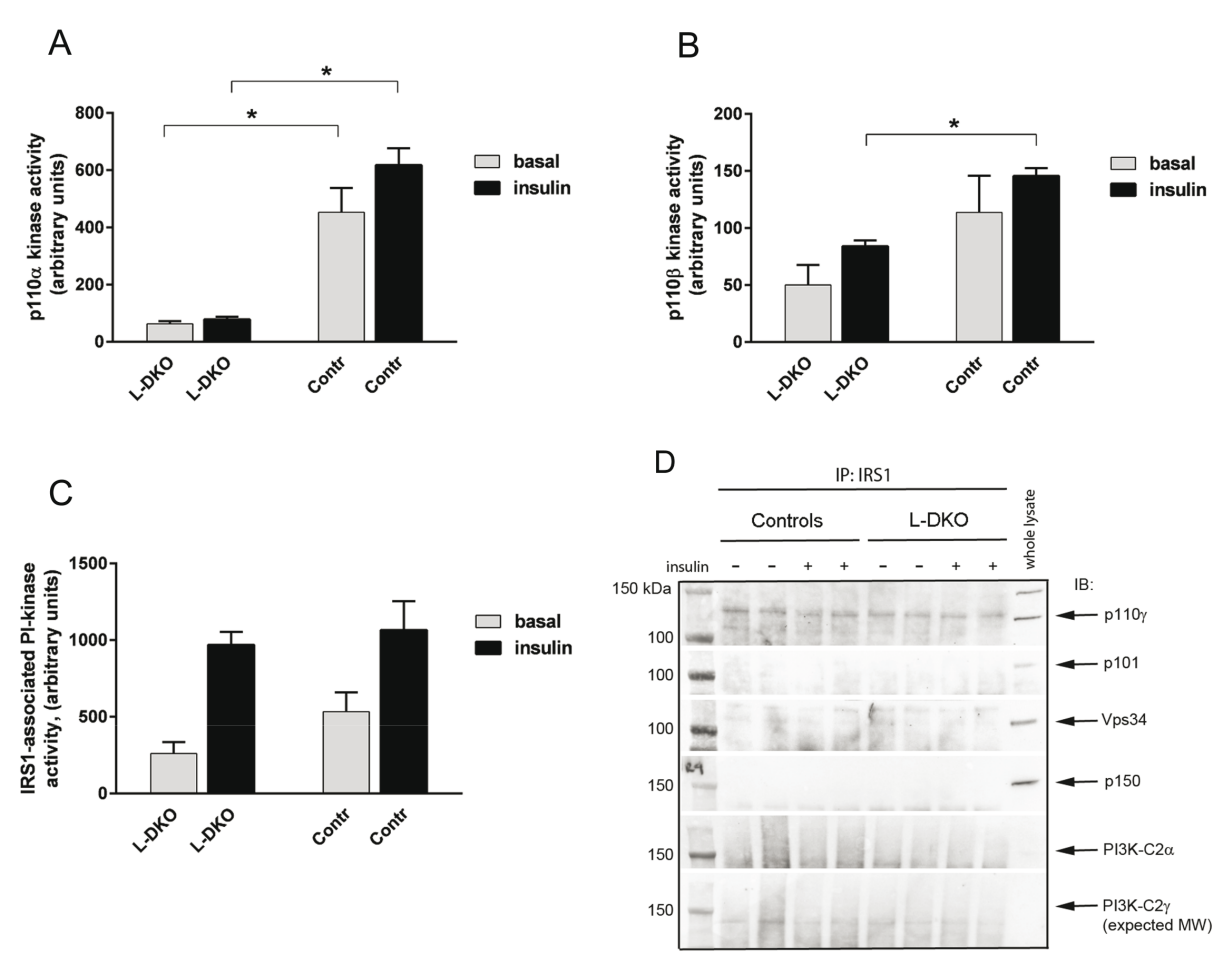
PI3K activity and assessment of the association of IRS1 with class IB-, class II- and class III-members of phosphoinositide 3-kinases. ( **A**) p110α lipid kinase activity, ( **B**) p110β lipid kinase activity, and ( **C**) IRS1-associated phosphatidylinositol (PI) kinase activity in L-DKO mice and controls were assessed by immunoprecipitation. ( **D**) Representative western blot of immunoprecipitation experiments with antibodies for IRS1 and subsequent immunoblotting with antibodies for p110γ, p101, Vps34, p150, PIK3-C2α and PIK3-C2γ. The whole-lysate reference sample was from an insulin-treated flox control mouse. IP = immunoprecipitation, IB = immunoblot. Basal condition is indicated with a minus (-) sign, insulin-treated condition is indicated with a plus (+) sign. Basal conditions refer to fasting of mice overnight and injection of saline through the vena cava. Insulin treatment refers to injection of 5 U of insulin through the vena cava 5 min prior to euthanization. Error bars indicate SEM (n = 4). *, p < 0.05 compared to controls.

As expected, p110α kinase activity, as assessed by the ability to add the 3’-phosphate group to phosphoinositides in p110α immunoprecipitates, was markedly decreased both in the basal- and insulin-stimulated states of the L-DKO mice compared to controls ( Figure 2A). Overall p110β kinase activity was much lower than p110α kinase activity in control mice, consistent with previous studies ^7, 27^, and was not changed in the basal state between controls and L-DKO mice ( Figure 2B). However, p110β kinase activity was significantly decreased in the insulin-stimulated state of the L-DKO mice ( Figure 2B), even though similar amounts of p110β protein were associated with IRS1 in controls and L-DKO mice ( Figure 1K and 1M). Surprisingly, IRS1-associated phosphatidylinositol kinase activity in response to insulin was intact in the L-DKO, despite lack of p110α kinase activity and decreased p110β activity ( Figure 2C).

### The intact IRS1-associated kinase activity in the L-DKO mice cannot be explained by compensatory upregulation of other classes of PI3K

To explore if other classes of phosphoinositide 3-kinases were responsible for the intact IRS1-associated activity, we investigated the association of IRS1 with class IB-, class II and class III members of phosphoinositide 3-kinases in liver lysates from controls and L-DKO mice. Class IB PI3K consists of the p110γ catalytic subunit and the p101 regulatory subunit. The amount of p101 was low in whole lysates and no p101 was associated with IRS1 in either control mice or L-DKO mice ( Figure 2D). p110γ was associated with IRS1, but the amounts were similar between controls and L-DKO mice ( Figure 2D). Class III PI3K catalytic subunit Vps34 and regulatory subunit p150 were present in whole lysates, but not associated with IRS1 in either control mice or L-DKO mice ( Figure 2D). Class II PI3K consists of only one subunit, but exists in several isoforms. The PI3K-C2α isoform is ubiquitously expressed, whereas PI3K-C2γ has been reported to have a more limited tissue distribution, including hepatocytes ^28, 29^. However, we detected only little or no PI3K-C2α or PI3K-C2γ in whole liver lysates or associated with IRS1 in controls or L-DKO mice ( Figure 2D). Thus, other classes of phosphoinositide 3-kinases did not compensate for the loss of p110α and p85α in the L-DKO mice and could not explain the intact IRS1-associated phosphatidylinositol kinase activity in the absence of p110α and p85α.

### L-DKO mice have decreased liver weight and WAT weight compared to controls

**Figure 3.**
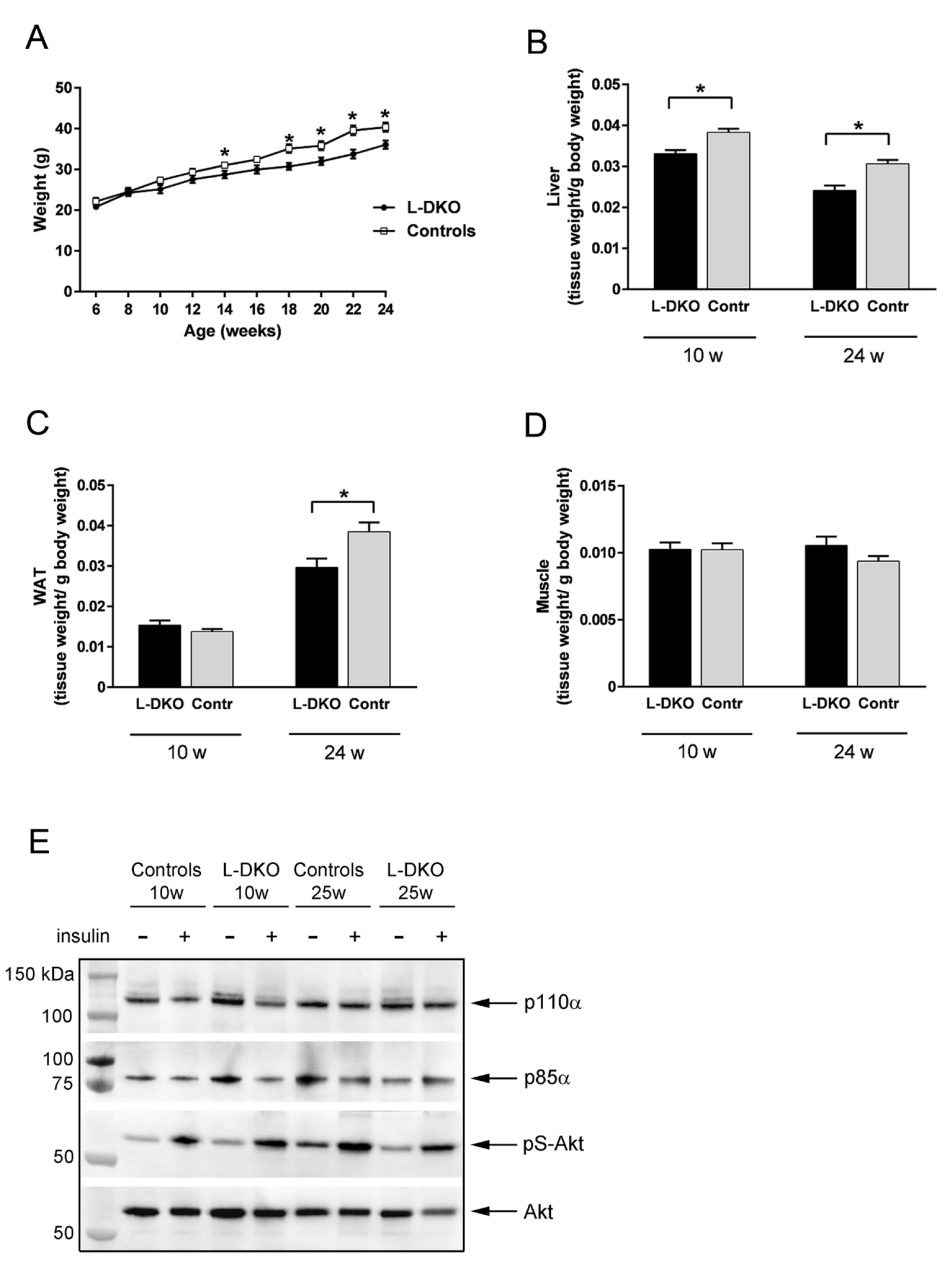
Body weight, tissue weights and adipose insulin signaling. ( **A**) whole body weight, ( **B**) liver weight, ( **C**) white adipose tissue (WAT) weight and ( **D**) muscle weight of 10 and 24 week old male L-DKO mice and controls. ( **E**) Representative western blot of p110α, p85α and phosphorylated Akt/PKB (Ser 473) in WAT of 10 and 25 week old male flox controls and L-DKO mice. Total Akt/PKB was used as a loading control. Basal condition is indicated with a minus (-) sign, insulin-treated condition is indicated with a plus (+) sign. Basal conditions refer to fasting of mice overnight and injection of saline through the vena cava. Insulin treatment refers to injection of 5 U of insulin through the vena cava 5 min prior to euthanization. Error bars indicate SEM (n = 8–17). *, p < 0.05 compared to controls. w = weeks.

On standard chow (4% fat content by weight, 12% by calories), L-DKO mice had similar body weights to the flox control mice until 10–12 weeks of age, after which the L-DKO mice showed slower weight gain compared to controls ( Figure 3A). At least part of this decrease was due to a decrease in liver weight. Thus, by 10 weeks of age, the ratio of liver weight to body weight in the L-DKO mice was decreased by 13% compared to control mice ( Figure 3B), whereas there was no difference in WAT weight or muscle weight ( Figures 3C and 3D). At 24 weeks of age, liver weight remained decreased (18%), and there was also a significant decreased WAT weight (22%) for the L-DKO mice compared to controls ( Figures 3B and 3C). To assess a possible change in insulin sensitivity in WAT, associated with the decreased WAT mass, we investigated insulin signaling mediators in this tissue. WAT p110α- and p85α expression was similar in controls and L-DKO mice as was Akt/PKB phosphorylation in response to insulin ( Figure 3E). There was no difference in insulin-stimulated Akt/PKB-activation at 10w compared to 25w in either controls or L-DKO mice ( Figure 3E).

### L-DKO mice have impaired glucose tolerance, but normal insulin tolerance and normal blood glucose levels

**Figure 4.**
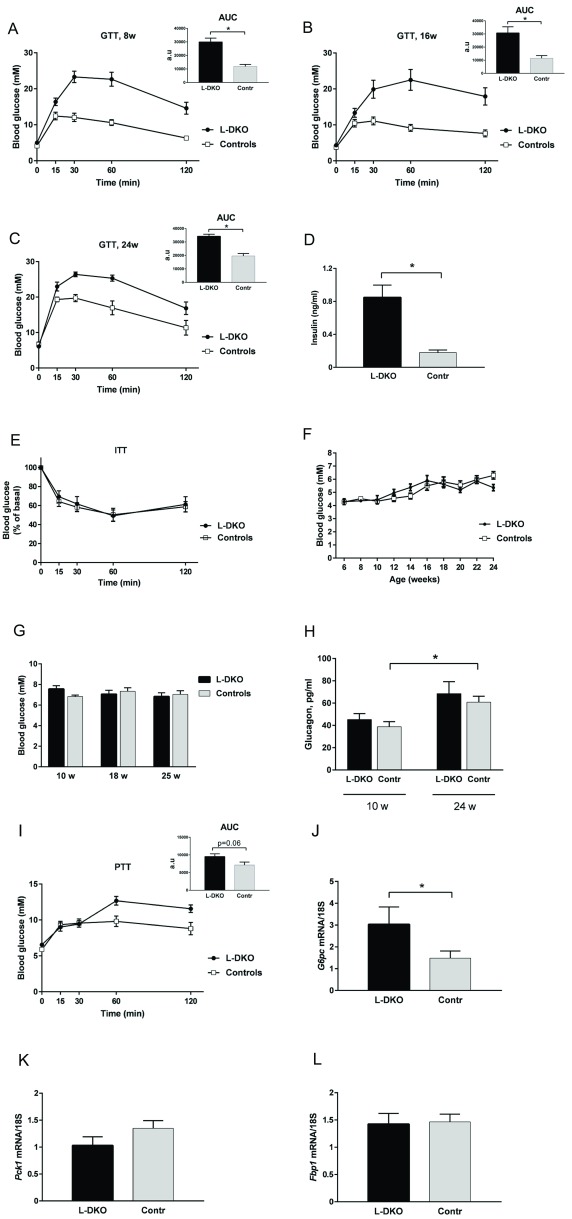
Glucose homeostasis. Glucose tolerance test at ( **A**) 8 weeks, ( **B**) 16 weeks and ( **C**) 24 weeks of age; ( **D**) fasting insulin levels at 10 weeks of age; ( **E**) insulin tolerance test at 19 weeks of age; ( **F**) fasting glucose levels; ( **G**) random fed glucose levels; ( **H**) fasting glucagon levels; ( **I**) pyruvate tolerance test at 15 weeks of age; ( **J**– **L**) hepatic mRNA expression of the gluconeogenic markers glucose 6-phosphatase ( *G6pc*), phosphoenolpyruvate carboxykinase ( *Pck1*) or fructose 1,6-bisphosphatase ( *Fbp1*). L-DKO mice and controls were given 2 g glucose/kg body weight intraperitoneally (i.p.) for the glucose tolerance test or 1.25 U insulin/kg body weight i.p. for the insulin tolerance test or 2 g pyruvate/kg body weight i.p. for the pyruvate tolerance test. Blood glucose was measured at 0, 15, 30, 60, and 120 min. Error bars indicate SEM (n =6–12). *, p < 0.05. The insets in the glucose tolerance test graphs and the pyruvate tolerance test graph show the area under the curve (AUC) with subtracted basal glucose values.

Despite similar body weight ( Figure 3A), L-DKO mice were severely glucose intolerant as early as at 8 weeks of age ( Figure 4A), and remained similarly glucose intolerant throughout the 24 week study ( Figures 4B and 4C). This was associated with markedly increased fasting insulin levels ( Figure 4D). However, somewhat surprisingly, the L-DKO mice showed a normal response to exogenous insulin during an intraperitoneal insulin tolerance test ( Figure 4E). Despite the markedly impaired glucose tolerance and marked hyperinsulinemia, fasting and random fed glucose levels in the L-DKO mice remained similar between controls and L-DKO mice ( Figures 4F and 4G). Circulating glucagon levels were also similar between L-DKO mice and controls both at 10 weeks of age and 24 weeks of age ( Figure 4H).

### L-DKO mice display normal hepatic glucose production

Increased gluconeogenesis is one of the hallmarks of hepatic insulin resistance in type 2 diabetes. To determine whether the impaired glucose tolerance in L-DKO mice was due to increased hepatic glucose output, we subjected these animals to a challenge with pyruvate, the major gluconeogenic substrate. Over the 120 min period following administration of pyruvate, there was a trend toward increased glucose levels in L-DKO mice compared to controls, but this was not statistically significant ( Figure 4I). This was associated with a significant change in hepatic gene expression of the gluconeogenic enzyme glucose 6-phosphatase ( *G6pc*) ( Figure 4J), whereas gene expression of the other key mediators of gluconeogenesis, phosphoenolpyruvate carboxykinase ( *Pck1*) and fructose 1,6-bisphosphatase ( *Fbp1*), remained similar between control mice and L-DKO mice ( Figures 4K and 4L).

Raw data for gene and protein expression shown in Figure 1 Click here for additional data file. Copyright: © 2018 Chaudhari A et al. 2018 Data associated with the article are available under the terms of the Creative Commons Zero "No rights reserved" data waiver (CC0 1.0 Public domain dedication).

Raw data for PI3K activity measurements and protein expression shown in Figure 2 Click here for additional data file. Copyright: © 2018 Chaudhari A et al. 2018 Data associated with the article are available under the terms of the Creative Commons Zero "No rights reserved" data waiver (CC0 1.0 Public domain dedication).

Raw data for body and tissue weights and protein expression shown in Figure 3 Click here for additional data file. Copyright: © 2018 Chaudhari A et al. 2018 Data associated with the article are available under the terms of the Creative Commons Zero "No rights reserved" data waiver (CC0 1.0 Public domain dedication).

Raw data for metabolic procedures and measurements and gene expression shown in Figure 4 Click here for additional data file. Copyright: © 2018 Chaudhari A et al. 2018 Data associated with the article are available under the terms of the Creative Commons Zero "No rights reserved" data waiver (CC0 1.0 Public domain dedication).

## Discussion

In this study, we investigated the impact of a combined deletion of p110α and p85α on insulin signaling and glucose homeostasis. For this purpose, we created mice with a liver-specific deletion of the major catalytic and major regulatory subunits of PI3K: *Pik3ca* and *Pik3r1*. We have previously shown that hepatic deletion of only p110α results in severe insulin resistance and impaired glucose tolerance, signifying that p110α is crucial for mediating insulin signaling ^7^. Moreover, mice deficient in all p85 isoforms in either muscle or liver exhibit severely impaired insulin signaling in these tissues ^14, 15^. The liver plays a crucial role in maintaining glucose homeostasis; we therefore hypothesized that deleting both these isoforms would result in severe and overt diabetes.

As expected, in the L-DKO mice, p110α catalytic activity was blunted and, as a result, the activation of the signal downstream of PI3K was markedly decreased. However, the L-DKO mice showed normal fasting and fed blood glucose levels throughout the study (24 weeks) and normal insulin tolerance. Glucose tolerance was impaired in the L-DKO mice and circulating insulin levels were markedly elevated, but to a degree similar to mice with only p110α deleted in the liver (L-p110α KO) ^7^. Surprisingly, despite abolished p110α activity, we observed an intact total IRS1-associated phosphatidylinositol (PI) kinase activity in the L-DKO mice. This finding was very different from what we previously observed in L-p110α KO mice, which only have p110α deleted in the liver ^7^. In the L-p110α KO mice, insulin-stimulated IRS1-associated PI kinase activity was markedly blunted ^7^. This suggests that it is the loss of the regulatory subunit that accounted for the preserved IRS1-associated activation, possibly by enabling other phosphatidylinositol kinases to bind to IRS1. However, there did not appear to be any compensatory effects of other known catalytic and regulatory class IA subunit isoforms for the action of insulin. Thus, we did not detect any differences in the IRS1-associated amounts of p110β, p110δ or p55γ in controls and L-DKO mice in response to insulin and only very small amounts of p85β associated with IRS1 in the L-DKO mice. Similarly, no evidence of compensatory effects by class IB, class II or class III members of phosphoinositide 3-kinases were found.

Previous studies by us and others, including our study of the L-p110α KO mice, have shown that p110β is unable to compensate for the loss of p110α ^7, 26, 30^. However, we observed similar amounts of IRS1-associated p110β in the L-DKO mice and controls despite overall total decreased levels of p110β in the liver. We therefore speculated that perhaps p110β activity was increased in response to insulin in the L-DKO mice compared to controls, which would explain the sustained IRS1-associated PI kinase activity. We found no difference in the p110β activity between the controls and the L-DKO mice in the basal state. In addition, the p110β kinase activity was significantly decreased, rather than increased, in the insulin-stimulated state of L-DKO mice. We thus conclude that the sustained IRS1-associated PI kinase activity in the L-DKO mice is not due to increased activity of p110β.

Although the amount of IRS1-associated p110β was similar in controls and L-DKO, the total amount of p110β was decreased in the L-DKO mice. We, and others, have previously reported that the dimeric interaction between the regulatory and the catalytic subunits results in stabilization of the subunits, whereas the monomeric forms are more readily subjected to degradation ^18, 20– 21^. Therefore, the absence of the major regulatory subunit p85α in the L-DKO mice probably subjects p110β to more rapid degradation. In this context, observing similar amounts of p110β associated with IRS1 in controls and L-DKO mice is somewhat surprising, but is likely due to stabilization of a fraction of p110β subunits by interaction with IRS1-associated p85β or p55γ.

Lack of the major regulatory subunit in the L-DKO mice, accompanied by absence of increased activation of p110β or compensatory increased expression of other phosphoinositide 3-kinases, suggests that presence of other classes of phosphatidylinositol kinases, perhaps PI4K and PI5K, account for the intact IRS1-associated kinase activity by directly binding to IRS1. PI4Ks have been described as mediators of endosomal trafficking from the Golgi and to be involved in EGF-stimulated phosphoinositide signaling ^31^. Type II PI4Ks interact with the EGF receptor, but they are not known to interact with IRS1. Type III PI4Ks are structurally related to PI3Ks, with a high degree of conservation between their catalytic domains and sensitivity to wortmannin ^31^. The isoform PI4KIIIα has been reported to be functionally connected to PI3K in FGF signaling during pectoral fin development in the zebra fish ^32^.

PI5K exists as two separate classes, PI(3)P5K and PI(4)P5K, phosphorylating the D5 position of the inositol ring of phosphatidylinositol 3-phosphate and phosphatidylinositol 4-phosphate, respectively. Of the PI(4)P5Ks, the isozyme PIP5Kc has been shown to respond to, and become phosphorylated by, various hormones and growth factors, such as EGF, and play a role in actin cytoskeletal reorganization, clathrin-dependent endocytosis, membrane ruffle formation, etc. ^33^. However, a direct effect on insulin signaling and interaction with IRSs by PIP5Kc has not been reported. PI(3)P5K, also called PIKfyve, and has been quite extensively studied and reported to be involved in membrane trafficking, stress- or hormone-induced signaling, ion channel activity, cytoskeletal dynamics, nuclear transport, gene transcription and cell cycle progression ^34^. Interestingly, PIKfyve is regulated by insulin, recruiting PIKfyve to inner membranes, where insulin receptor and IRSs are also found ^35^, and co-precipitates with p110 and p85 subunits in 3T3-L1 adipocytes ^36^. Thus, PIKfyve appears to be a possible contributor to the sustained IRS1-associated kinase activity in the L-DKO mice. However, PIKfyve expression has been reported to be rather tissue specific, mainly expressed in adipose tissue, muscle and brain ^37, 38^ and expression in the liver appears low ^37, 38^. A more extensive follow-up investigation of the various phosphoinositides in the L-DKO livers compared to controls may help elucidating the phosphatidylinositol kinase responsible for the sustained IRS1-associated kinase activity in the L-DKO mice.

The absence of both p85α and p110α, as seen in the L-DKO mice, would logically result in a very severely impaired metabolic phenotype. The L-DKO mice had markedly elevated circulating insulin levels, impaired glucose tolerance and showed a trend toward increased rates of hepatic glucose output when given pyruvate. However, fasted and fed glucose levels were not different between controls and L-DKO mice, i.e., randomly fed L-DKO mice were not diabetic and insulin tolerance tests were normal. Part of this protection might be the fact that L-DKO mice had less accumulation of WAT with age. In addition, it is possible that the high circulating insulin levels reflect an impaired hepatic insulin clearance rather than insulin resistance in the muscle, which would explain the paradoxical normal insulin tolerance. Interestingly, body weight, WAT weight and hepatic glucose output were significantly increased and insulin tolerance severely impaired in L-p110α KO mice ^7^, which lack only p110α in liver, demonstrating that L-DKO mice showed an overall less severe metabolic phenotype compared to L-p110α KO mice.

In summary, deletion of hepatic p110α and p85α results in an impaired insulin signal and impaired glucose homeostasis, but shows an overall less severe metabolic phenotype compared to mice with only p110α deleted in the liver. Although other PI3Ks were unable to compensate for the loss of p110α and p85α, IRS1-associated phosphatidylinositol kinase activity was surprisingly still intact, possibly due to interaction of IRS1 with other classes of phosphatidylinositol kinases.

**Abbreviations:** Fbp, fructose-1,6-bisphosphatase; G6pc, glucose-6-phosphatase; GTT, glucose tolerance test; ITT, insulin tolerance test; L-DKO, liver double knockout; Pck, phosphoenolpyruvate carboxykinase; PI3K, phosphatidylinositol-4,5-bisphosphate 3-kinase; PKB, protein kinase B; PTT, pyruvate tolerance test; WAT, white adipose tissue

## Data availability

The data referenced by this article are under copyright with the following copyright statement: Copyright: © 2018 Chaudhari A et al.

Data associated with the article are available under the terms of the Creative Commons Zero "No rights reserved" data waiver (CC0 1.0 Public domain dedication). 

**Dataset 1:** Raw data for gene and protein expression shown in Figure 1. DOI, 10.5256/f1000research.12418.d205909 ^39^

**Dataset 2:** Raw data for PI3K activity measurements and protein expression shown in Figure 2. DOI, 10.5256/f1000research.12418.d175494 ^40^

**Dataset 3:** Raw data for body and tissue weights and protein expression shown in Figure 3. DOI, 10.5256/f1000research.12418.d175495 ^41^

**Dataset 4:** Raw data for metabolic procedures and measurements and gene expression shown in Figure 4. DOI, 10.5256/f1000research.12418.d205910 ^42^

## References

[1] Engelman JA Luo J Cantley LC: The evolution of phosphatidylinositol 3-kinases as regulators of growth and metabolism. *Nat Rev Genet.* 2006;7(8):606–619. 10.1038/nrg1879 16847462

[2] Taniguchi CM Emanuelli B Kahn CR: Critical nodes in signalling pathways: insights into insulin action. *Nat Rev Mol Cell Biol.* 2006;7(2):85–96. 10.1038/nrm1837 16493415

[3] Fransson S Uv A Eriksson H: p37δ is a new isoform of PI3K p110δ that increases cell proliferation and is overexpressed in tumors. *Oncogene.* 2012;31(27):3277–3286. 10.1038/onc.2011.492 22020336

[4] Ejeskär K Vickes O Kuchipudi A: The Unique Non-Catalytic C-Terminus of P37delta-PI3K Adds Proliferative Properties *In Vitro* and *In Vivo*. *PLoS One.* 2015;10(5):e0127497. 10.1371/journal.pone.0127497 26024481 PMC4449119

[5] Foukas LC Claret M Pearce W: Critical role for the p110alpha phosphoinositide-3-OH kinase in growth and metabolic regulation. *Nature.* 2006;441(7091):366–370. 10.1038/nature04694 16625210

[6] Knight ZA Gonzalez B Feldman ME: A pharmacological map of the PI3-K family defines a role for p110alpha in insulin signaling. *Cell.* 2006;125(4):733–747. 10.1016/j.cell.2006.03.035 16647110 PMC2946820

[7] Sopasakis VR Liu P Suzuki R: Specific Roles of the p110alpha Isoform of Phosphatidylinsositol 3-Kinase in Hepatic Insulin Signaling and Metabolic Regulation. *Cell Metab.* 2010;11(3):220–230. 10.1016/j.cmet.2010.02.002 20197055 PMC3144706

[8] Ciraolo E Iezzi M Marone R: Phosphoinositide 3-kinase p110beta activity: key role in metabolism and mammary gland cancer but not development. *Sci Signal.* 2008;1(36):ra3. 10.1126/scisignal.1161577 18780892 PMC2694958

[9] Jia S Liu Z Zhang S: Essential roles of PI(3)K-p110beta in cell growth, metabolism and tumorigenesis. *Nature.* 2008;454(7205):776–779. 10.1038/nature07091 18594509 PMC2750091

[10] Patton DT Garçon F Okkenhaug K: The PI3K p110delta controls T-cell development, differentiation and regulation. *Biochem Soc Trans.* 2007;35(Pt 2):167–171. 10.1042/BST0350167 17371229

[11] Bilancio A Okkenhaug K Camps M: Key role of the p110delta isoform of PI3K in B-cell antigen and IL-4 receptor signaling: comparative analysis of genetic and pharmacologic interference with p110delta function in B cells. *Blood.* 2006;107(2):642–650. 10.1182/blood-2005-07-3041 16179367

[12] Eickholt BJ Ahmed AI Davies M: Control of axonal growth and regeneration of sensory neurons by the p110delta PI 3-kinase. *PLoS One.* 2007;2(9):e869. 10.1371/journal.pone.0000869 17846664 PMC1959241

[13] Ueki K Algenstaedt P Mauvais-Jarvis F: Positive and negative regulation of phosphoinositide 3-kinase-dependent signaling pathways by three different gene products of the p85alpha regulatory subunit. *Mol Cell Biol.* 2000;20(21):8035–8046. 10.1128/MCB.20.21.8035-8046.2000 11027274 PMC86414

[14] Taniguchi CM Kondo T Sajan M: Divergent regulation of hepatic glucose and lipid metabolism by phosphoinositide 3-kinase via Akt and PKClambda/zeta. *Cell Metab.* 2006;3(5):343–353. 10.1016/j.cmet.2006.04.005 16679292

[15] Luo J Sobkiw CL Hirshman MF: Loss of class I _A_ PI3K signaling in muscle leads to impaired muscle growth, insulin response, and hyperlipidemia. *Cell Metab.* 2006;3(5):355–366. 10.1016/j.cmet.2006.04.003 16679293

[16] Fruman DA Mauvais-Jarvis F Pollard DA: Hypoglycaemia, liver necrosis and perinatal death in mice lacking all isoforms of phosphoinositide 3-kinase p85 alpha. *Nat Genet.* 2000;26(3):379–382. 10.1038/81715 11062485

[17] Mauvais-Jarvis F Ueki K Fruman DA: Reduced expression of the murine p85alpha subunit of phosphoinositide 3-kinase improves insulin signaling and ameliorates diabetes. *J Clin Invest.* 2002;109(1):141–149. 10.1172/JCI13305 11781359 PMC150818

[18] Terauchi Y Tsuji Y Satoh S: Increased insulin sensitivity and hypoglycaemia in mice lacking the p85 alpha subunit of phosphoinositide 3-kinase. *Nat Genet.* 1999;21(2):230–235. 10.1038/6023 9988280

[19] Brachmann SM Ueki K Engelman JA: Phosphoinositide 3-kinase catalytic subunit deletion and regulatory subunit deletion have opposite effects on insulin sensitivity in mice. *Mol Cell Biol.* 2005;25(5):1596–1607. 10.1128/MCB.25.5.1596-1607.2005 15713620 PMC549361

[20] Barbour LA Mizanoor Rahman S Gurevich I: Increased P85alpha is a potent negative regulator of skeletal muscle insulin signaling and induces *in vivo* insulin resistance associated with growth hormone excess. *J Biol Chem.* 2005;280(45):37489–37494. 10.1074/jbc.M506967200 16166093

[21] Ueki K Fruman DA Brachmann SM: Molecular balance between the regulatory and catalytic subunits of phosphoinositide 3-kinase regulates cell signaling and survival. *Mol Cell Biol.* 2002;22(3):965–977. 10.1128/MCB.22.3.965-977.2002 11784871 PMC133541

[22] Yu J Zhang Y McIlroy J: Regulation of the p85/p110 phosphatidylinositol 3'-kinase: stabilization and inhibition of the p110alpha catalytic subunit by the p85 regulatory subunit. *Mol Cell Biol.* 1998;18(3):1379–1387. 10.1128/MCB.18.3.1379 9488453 PMC108851

[23] Luo J Field SJ Lee JY: The p85 regulatory subunit of phosphoinositide 3-kinase down-regulates IRS-1 signaling via the formation of a sequestration complex. *J Cell Biol.* 2005;170(3):455–464. 10.1083/jcb.200503088 16043515 PMC2171479

[24] Taniguchi CM Tran TT Kondo T: Phosphoinositide 3-kinase regulatory subunit p85alpha suppresses insulin action via positive regulation of PTEN. *Proc Natl Acad Sci U S A.* 2006;103(32):12093–12097. 10.1073/pnas.0604628103 16880400 PMC1524929

[25] Taniguchi CM Aleman JO Ueki K: The p85alpha regulatory subunit of phosphoinositide 3-kinase potentiates c-Jun N-terminal kinase-mediated insulin resistance. *Mol Cell Biol.* 2007;27(8):2830–2840. 10.1128/MCB.00079-07 17283057 PMC1899914

[26] Zhao JJ Cheng H Jia S: The p110alpha isoform of PI3K is essential for proper growth factor signaling and oncogenic transformation. *Proc Natl Acad Sci U S A.* 2006;103(44):16296–16300. 10.1073/pnas.0607899103 17060635 PMC1637576

[27] Chaudhari A Krumlinde D Lundqvist A: p110α Hot Spot Mutations E545K and H1047R Exert Metabolic Reprogramming Independently of p110α Kinase Activity. *Mol Cell Biol.* 2015;35(19):3258–3273. 10.1128/MCB.00471-15 26169833 PMC4561724

[28] Domin J Pages F Volinia S: Cloning of a human phosphoinositide 3-kinase with a C2 domain that displays reduced sensitivity to the inhibitor wortmannin. *Biochem J.* 1997;326(Pt 1):139–147. 10.1042/bj3260139 9337861 PMC1218647

[29] Rozycka M Lu YJ Brown RA: cDNA cloning of a third human C2-domain-containing class II phosphoinositide 3-kinase, PI3K-C2gamma, and chromosomal assignment of this gene (PIK3C2G) to 12p12. *Genomics.* 1998;54(3):569–574. 10.1006/geno.1998.5621 9878262

[30] Bi L Okabe I Bernard DJ: Proliferative defect and embryonic lethality in mice homozygous for a deletion in the p110alpha subunit of phosphoinositide 3-kinase. *J Biol Chem.* 1999;274(16):10963–10968. 10.1074/jbc.274.16.10963 10196176

[31] Balla A Balla T: Phosphatidylinositol 4-kinases: old enzymes with emerging functions. *Trends Cell Biol.* 2006;16(7):351–361. 10.1016/j.tcb.2006.05.003 16793271

[32] Ma H Blake T Chitnis A: Crucial role of phosphatidylinositol 4-kinase IIIalpha in development of zebrafish pectoral fin is linked to phosphoinositide 3-kinase and FGF signaling. *J Cell Sci.* 2009;122(Pt 23):4303–4310. 10.1242/jcs.057646 19887586 PMC2779132

[33] Funakoshi Y Hasegawa H Kanaho Y: Regulation of PIP5K activity by Arf6 and its physiological significance. *J Cell Physiol.* 2011;226(4):888–895. 10.1002/jcp.22482 20945365

[34] Jin N Lang MJ Weisman LS: Phosphatidylinositol 3,5-bisphosphate: regulation of cellular events in space and time. *Biochem Soc Trans.* 2016;44(1):177–184. 10.1042/BST20150174 26862203 PMC4836390

[35] Shisheva A Rusin B Ikonomov OC: Localization and insulin-regulated relocation of phosphoinositide 5-kinase PIKfyve in 3T3-L1 adipocytes. *J Biol Chem.* 2001;276(15):11859–11869. 10.1074/jbc.M008437200 11112776

[36] Sbrissa D Ikonomov O Shisheva A: Selective insulin-induced activation of class I _A_ phosphoinositide 3-kinase in PIKfyve immune complexes from 3T3-L1 adipocytes. *Mol Cell Endocrinol.* 2001;181(1–2):35–46. 10.1016/S0303-7207(01)00539-1 11476939

[37] Shisheva A Sbrissa D Ikonomov O: Cloning, characterization, and expression of a novel Zn ^2+^-binding FYVE finger-containing phosphoinositide kinase in insulin-sensitive cells. *Mol Cell Biol.* 1999;19(1):623–634. 10.1128/MCB.19.1.623 9858586 PMC83920

[38] Ikonomov OC Sbrissa D Delvecchio K: Muscle-specific *Pikfyve* gene disruption causes glucose intolerance, insulin resistance, adiposity, and hyperinsulinemia but not muscle fiber-type switching. *Am J Physiol Endocrinol Metab.* 2013;305(1):E119–131. 10.1152/ajpendo.00030.2013 23673157 PMC3725567

[39] Chaudhari A Ejeskär K Wettergren Y: Dataset 1 in: Hepatic deletion of p110α and p85α results in insulin resistance despite sustained IRS1-associated phosphatidylinositol kinase activity. *F1000Research.* 2018 Data Source 10.12688/f1000research.12418.1 PMC6020741 29983910

[40] Chaudhari A Ejeskär K Wettergren Y: Dataset 2 in: Hepatic deletion of p110a and p85a results in insulin resistance despite sustained IRS1-associated phosphatidylinositol kinase activity. *F1000Research.* 2017 Data Source 10.12688/f1000research.12418.1 PMC6020741 29983910

[41] Chaudhari A Ejeskär K Wettergren Y: Dataset 3 in: Hepatic deletion of p110a and p85a results in insulin resistance despite sustained IRS1-associated phosphatidylinositol kinase activity. *F1000Research.* 2017 Data Source 10.12688/f1000research.12418.1 PMC6020741 29983910

[42] Chaudhari A Ejeskär K Wettergren Y: Dataset 4 in: Hepatic deletion of p110α and p85α results in insulin resistance despite sustained IRS1-associated phosphatidylinositol kinase activity. *F1000Research.* 2018 Data Source 10.12688/f1000research.12418.1 PMC6020741 29983910

