# Supplementary material for: Hepatic deletion of p110α and p85α results in insulin resistance despite sustained IRS1-associated phosphatidylinositol kinase activity
Source: F1000Res. 2018 Jun 5;6:1600. Originally published 2017 Aug 30. [Version 2] doi: 10.12688/f1000research.12418.2 (PMC6020741; doi:10.12688/f1000research.12418.2)
Supplement: Peer review history

# Peer review: Referee response for version 2

**DOI:** 10.5256/f1000research.16404.r34701

**Published:** 2018-07-05

**Authors:** Lily Q. Dong

**Affiliations:** 1 Department of Cell Systems & Anatomy, University of Texas Health at San Antonio, San Antonio, TX, USA

The revised version is acceptable.

I have read this submission. I believe that I have an appropriate level of expertise to confirm that it is of an acceptable scientific standard.

# Peer review: Referee response for version 2

**DOI:** 10.5256/f1000research.16404.r34702

**Published:** 2018-06-28

**Authors:** Richard Z. Lin, Lisa Ballou

**Affiliations:** 1 Department of Physiology and Biophysics, Stony Brook School of Medicine, Stony Brook, NY, USA

The manuscript is still not fully acceptable. Please see our comments below:

 Fig. 2.: The conclusion that p110β is not responsible for at least some of the the IRS1–associated kinase activity in L-DKO mice is still not well supported by the evidence. The western blots in Fig. 1K are not convincing, and the quantified western blots in Fig. 1M have large error bars. In Fig. 2B, the p110beta antibody used for IP might be much less efficient than the IRS1 antibody in pulling down the enzyme. The IRS1 IPs should be assayed in the presence and absence of TGX-221, which is highly selective for p110beta in vitro. This assay is not more complicated than the ones already shown in the figure. 

 Fig. 3: Please indicate in the legend which muscles and WAT depots were analyzed.

We have read this submission. We believe that we have an appropriate level of expertise to confirm that it is of an acceptable scientific standard, however we have significant reservations, as outlined above.

# Peer review: Referee response for version 2

**DOI:** 10.5256/f1000research.16404.r34700

**Published:** 2018-06-25

**Authors:** James R. Woodgett, Prital Patel

**Affiliations:** 1 Lunenfeld-Tanenbaum Research Institute, Sinai Health System & University of Toronto, Toronto, ON, Canada

The revised version fully satisfies my comments previously made with respect to the original submission.

We have read this submission. We believe that we have an appropriate level of expertise to confirm that it is of an acceptable scientific standard.

# Peer review: Referee response for version 1

**DOI:** 10.5256/f1000research.13447.r27377

**Published:** 2017-11-15

**Authors:** Richard Z. Lin, Lisa Ballou

**Affiliations:** 1 Department of Physiology and Biophysics, Stony Brook School of Medicine, Stony Brook, NY, USA

**General Comments:**

 The authors made a very interesting observation that L-DKO mice, lacking hepatic p110α and p85α, have a milder metabolic phenotype than mice lacking only hepatic p110α. These mice have normal fasting blood glucose levels but not surprisingly they are glucose intolerant. Interestingly, the liver and WAT weights were reduced. Liver triglyceride levels were not measured to determine if the fat content of the liver was also reduced. Metabolic cage measurements would be revealing to rule in or out changes in energy expenditure of the animals. It would also be informative to know if a high-fat diet causes L-DKO animals to develop fasting hyperglycemia and/or if the fatty liver associated with this type of diet is accentuated or attenuated.

 In general, the results from the insulin signaling experiments are not convincing. It is unclear why the authors concluded that the residual signaling to Akt is not due to Class I PI3Ks. IRS-1 pull-downs from L-DKO liver have substantial PI kinase activity. Better experiments are needed to resolve this research question (see comment below).

**Specific Comments:**

 Figures 1F & 1G, Figure 2 and Figure 3E. (a) The dose of insulin used to study insulin signaling was extremely high. 5 U injected into a 30 g mouse is 167 U/kg body weight. Compare that to the 1.25 U/kg body weight used for the insulin tolerance test. Some of the signaling changes could be due to activation of an unusually high percentage of insulin receptors or insulin activation of IGF1 receptors that would not occur under physiological conditions. Attempts to explain the pathophysiological phenotypes of the L-DKO mouse based on the signaling results is therefore problematic. (b) The legends say that “Basal conditions refer to fasting of mice overnight.” Were the control mice anesthetized and injected ivc with saline, as stated under Methods? If so, the legends could be more specific.

 Figure 1F. Total Akt and total p70S6K should be shown.

 Figure 1G. (a) Why did insulin treatment of control mice not increase the amount of p85α (or other regulatory subunit) associated with IRS-1? (b) Use of a pan p85 antibody to estimate changes in p85β expression is not convincing. Lack of a good mouse p85β antibody is a limitation to the study. (c) The conclusion that similar amounts of IRS-1–bound p110β were pulled down in control and L-DKO mice is not convincing based on the blot.

 Figure 2. The conclusion that p110β and the other PI3Ks examined by western blotting are not responsible for the IRS-1–associated kinase activity is premature. The IPs should be assayed in the presence and absence of selective inhibitors to determine the contribution of p110β and the other enzymes to the PI kinase activity. Considering that hepatocytes make up 75-80% of the cell types in the liver, it is also possible that some of the kinase activity in the IRS-1 IP comes from p110α in the 20-25% of cells that are not targeted by albumin-Cre.

 Figure 3. Histological examination of the livers should be included. Liver triglyceride levels should be measured.

 Figures 3C & 3D. The specific WAT depots and muscles examined should be identified.

 Figure 4A-D. Fasting insulin is higher in the L-DKO mice at 10 weeks of age. Presumably, this is why these mice are euglycemic. Insulin levels should be measured before and after the glucose challenge with mice in one of the GTT age groups. This experiment will assess if the glucose intolerance is due to inability of the L-DKO mice to mount a further insulin response.

We have read this submission. We believe that we have an appropriate level of expertise to confirm that it is of an acceptable scientific standard, however we have significant reservations, as outlined above.

# Response

**Published:** 2018-05-21

**Authors:** Victoria Rotter Sopasakis

**Affiliations:** University of Gothenburg, Sweden

Response to general comments:

 We agree with the reviewers that all these studies would be very interesting, but would take several years to complete and is outside the scope of this particular study.

 Response to specific comments:

 1a. The dose of insulin used is what is normally used for these types of experiments. If the reviewers would be right, i.e unusually high activation of insulin would occur, this would be the case in all our other similar studies (and all other scientists’ studies who are doing similar experiments), for example in the study where we knocked out p110a in the liver. Since this is not the case, we don’t believe that this a major problem for the interpretation of our data.

 1b. Yes, the control mice were anesthetized and injected ivc with saline (basal conditions) or insulin. This has been added to the figure legends as required.

 2. Total Akt and total p70S6K blots have been added to Figure 1F.

 3a. We have added more experiments to this figure (n=4) and made quantitative graphs of the results. The amount of p85a associated with IRS1 still remains similar between basal and insulin-stimulated conditions, whereas the p85-pan antibody shows increased amounts of total p85 in response to insulin (figures 1N and 1O).

 3b. We have previously (before submitting the manuscript) tried various different p85b antibodies without any success. Some work well for human tumor tissue for example, but give no band at all for our mouse liver lysates. Other antibodies we tried fail to deliver a band for both human and mouse tissues. This is the reason we chose to use the p85-pan antibody to indirectly show p85b protein expression. Since both Dr Woodgett and Drs Lin and Ballou requested a p85b antibody we have tried again to make various p85b antibodies on the market to work for our liver lysates and have spent months now trying to optimize the western blot conditions for these antibodies. We are not satisfied with the results. The only conclusion we can draw is that either 1) the p85b antibodies available on the market simply does not work well for mouse tissue or 2) there is not much p85b expressed in mouse liver. Based on the p85-pan westerns that we performed as well as previous studies including Taniguchi et al, we believe that there simply is not that much p85b expressed in mouse liver.

 3c. More experiments have been added (n=4) and quantifying graphs are now included in the figure (figure 1M).

 4. These types of experiments are all very interesting. However, they are complicated, particularly considering that inhibitors are never 100% selective and specific. We therefore question the gain of these experiments in relation to the work and resources that have to be invested to complete them.

 5.  Histological experiments and liver triglyceride level measurements are outside the scope of this study.

 6. These types of experiments would not lead to increased understanding of the underlying mechanisms of our data. We therefore question the gain of these experiments in relation to the work and resources that have to be invested to complete them. 

 7. It is quite possible that the L-DKO mice are not able to further increase the insulin secretion is response to the glucose load in the GTT considering the very high insulin levels these mice have normally. We have added a glucagon ELISA (new figure 4H) that shows normal glucagon levels for DKO mice both at 10 w of age and 24 w of age. The normal glucagon levels and the very high insulin levels in the DKO mice indicates that the pancreas is working normally in these mice and the underlying mechanism for the glucose intolerance is therefore not likely at the level of the pancreas.

# Peer review: Referee response for version 1

**DOI:** 10.5256/f1000research.13447.r27376

**Published:** 2017-11-08

**Authors:** Lily Q. Dong

**Affiliations:** 1 Department of Cell Systems & Anatomy, University of Texas Health at San Antonio, San Antonio, TX, USA

Chaudhari et al. reported the physiologic roles of hepatic p110a and p85a in regulating insulin signaling and glucose homeostasis. They concluded that hepatic deletion of p110α and p85α (L-DKO) led to mild metabolic phenotype of the L-DKO mice and proposed that lipid kinases other than PI3Ks might compensate for the loss of p110α/p85α by signaling through unknown signaling pathway other than Akt signaling. Since deletion of p85α leads to improved insulin sensitivity and deletion of p110α leads to impaired insulin signaling and insulin resistance, it is interesting to learn that L-DKO mice showed an overall less severe metabolic phenotype compared to the L-p110a KO mice. 

1. Although the expression levels of p110b were decreased in the liver of L-DKO mice (Fig. 1E), IRS1-associated p110b levels were comparable between the control and KO mice (Fig. 1G). In addition, notable levels of p110b activity were maintained in the L-DKO mice and insulin treatment can stimulate the activity although it is 50% compared to the control (Fig. 2B). It is unclear why the authors concluded that the intact IRS1-associated kinase activity in the KO mice are not from the remaining p110b. 
2. The KO mice showed significantly impaired glucose tolerance and markedly increased fasting insulin levels (Fig. 4). What were the pancreatic phenotype of the KO mice? What were the levels of glucagon in the KO mice?

I have read this submission. I believe that I have an appropriate level of expertise to confirm that it is of an acceptable scientific standard, however I have significant reservations, as outlined above.

# Response

**Published:** 2018-05-21

**Authors:** Victoria Rotter Sopasakis

**Affiliations:** University of Gothenburg, Sweden

1. We don’t believe that the intact IRS1-associated kinase activity in the DKO mice are from the remaining p110b for the following reasons:

 The total degree of kinase activity is very much smaller for p110b compared to p110a. This is an observation we have made over and over again for liver lysates in response to insulin in several different studies (the a.u numbers of the y-axis in the graphs in figure 2 demonstrates this to some extent). However, we still wanted to be sure that we were correct to rule out a role of p110b since we observed similar amounts of p110b bound to IRS1 in the DKO and controls. It was therefore important to look at the kinase activity associated with IRS1. The p110b-associated kinase activity in response to insulin was significantly decreased in the DKO mice compared to controls (figure 2b). These two observations (   1) very much smaller degree of p110b activity over all compared to p110a kinase activity and 2) decreased IRS1-associated p110b activity in the DKO in response to insulin) makes it highly inlikely that the sustained IRS1-associated kinase activity is due to compensatory levels of p110b kinase activity).

 2. We have performed a glucagon ELISA assay for the mice (new figure 4H). There were no differences in glucagon serum levels between DKO mice and controls at either 10 weeks of age or 24 weeks of age. The high fasting insulin levels in the DKO mice suggest that insulin production and secretion by the pancreas is intact and since no differences in glucagon secretion was observed even at older age we conclude that the pancreatic phenotype is likely very similar between DKO mice and controls.

# Peer review: Referee response for version 1

**DOI:** 10.5256/f1000research.13447.r25513

**Published:** 2017-09-14

**Authors:** James R. Woodgett, Prital Patel

**Affiliations:** 1 Lunenfeld-Tanenbaum Research Institute, Sinai Health System & University of Toronto, Toronto, ON, Canada

In this study, the authors generated a liver-specific double knockout of the regulatory and catalytic subunit of Class 1A PI3K. Previously, single inactivation of either the catalytic art regulatory subunits had been shown to result in opposite effects on insulin resistance vs sensitivity. The authors demonstrated through *in vivo* studies in male mice (129Sv-C57Bl/6) that defects in glucose tolerance as well as changes in liver, fat and body weight occur upon deletion of both the regulatory PIK3R1(p85α) and catalytic subunit PIK3CA (p110α) but retained normal production of gulches from the liver.

**Specific Comments:**

1. Figure 1E (page 6): the authors state (on page 5) that low levels of p85β were detected in the L-DKO mice compared to controls. This was indirectly demonstrated through the use of a pan-p85 antibody that detects both p85α and p85β. Mice with liver-specific deletion of p85α on a p85β null background have been previously characterized (Taniguchi *et al.*). There, the overall levels of p85β detected utilizing a pan p85β antibody in a lysate derived from a liver that was wild type for p85β and null for p85α was minimal. Therefore, it is possible that the overall protein content reduction observed using the pan p85 antibody in Figure1E may be explained by the deletion of p85α from the liver, and may not be due to any change in p85β levels. To directly compare wild type and L-DKO samples, a specific p85β antibody should be used. Loading controls should also be provided for some panels in Figure 1.
2. Figure 1F to show impaired activation, total AKT blots should be provided (similar to Figure 3E). Total levels of p70S6K also need to be measured. In Figure 1G, the authors state that although p110β levels are reduced, the amount associated with IRS-1 is unchanged. However, due to the variability across samples in both control and L-DKO, and the overall poor quality of the blot, these conclusions don’t inspire confidence. These data should be quantitated. Additionally, the p110β band on the last lane appears to run slightly higher compared to the rest of the bands and the control raising possible questions over the specificity of the p110β antibody used for IP. Are similar results observed using a distinct antibody? In line with the comments in point 1, the authors state that the overall amount of p85β associated IRS-1 does not change. This conclusion requires use of a p85β selective antibody.
3. As noted by the authors, there is known complexity between the structural roles of the various PI3K subunits and isoforms, with effects on protein stability and access to IRS-1 binding sites. There appears, as noted above, to be significant variation in levels of the untargetted subunits across the 2 typically shown animals (e.g. Figure 1G). If other animals were measured, a composite of the levels (e.g. n=4) would provide much better insight into the levels (if any) of compensation, allowing a clearer picture of what may be underlying the glucose metabolic effects. It is also possible there was downstream adaptation given the IRS-1 is negatively regulated by PI3K signaling (and appears somewhat higher in the DKOs (Figure 1G).
4. For the PI3K lipid kinase activity measurements (chromatographic separation, Figure 2 shows the quantitation), how were the separate plates normalized? 

Minor grammatical error:

 Page 11, second paragraph line 10- “and” is added in error. 

 *Taniguchi, C. M., T. Kondo, M. Sajan, J. Luo, R. Bronson, T. Asano, R. Farese, L. C. Cantley and C. R. Kahn (2006). "Divergent regulation of hepatic glucose and lipid metabolism by phosphoinositide 3-kinase via Akt and PKClambda/zeta." Cell Metab **3**(5): 343-353.

We have read this submission. We believe that we have an appropriate level of expertise to confirm that it is of an acceptable scientific standard, however we have significant reservations, as outlined above.

# Response

**Published:** 2018-05-21

**Authors:** Victoria Rotter Sopasakis

**Affiliations:** University of Gothenburg, Sweden

1. We totally agree with the Dr Woodgett’s point here and it is essentially what we tried to convey in the manuscript, but to make it clearer we have added an extra sentence including the reference to Taniguchi et al (page 9, row 7).

 We have previously (before submitting the manuscript) tried various different p85b antibodies without any success. Some work well for human tumor tissue for example, but give no band at all for our mouse liver lysates. Other antibodies we tried fail to deliver a band for both human and mouse tissues. This is the reason we chose to use the p85-pan antibody to indirectly show p85b protein expression. Since both Dr Woodgett and Drs Lin and Ballou requested a p85b antibody we have tried again to make various p85b antibodies on the market to work for our liver lysates and have spent months now trying to optimize the western blot conditions for these antibodies. We are not satisfied with the results. The only conclusion we can draw is that either 1) the p85b antibodies available on the market simply does not work well for mouse tissue or 2) there is not much p85b expressed in mouse liver (just as Dr Woodgett points out above). Based on the p85-pan westerns that we performed as well as previous studies including Taniguchi et al, to which Dr Woodgett referred, we believe that there simply is not that much p85b expressed in mouse liver.

 Loading controls have been added to figure 1 E and 1F, including Akt and p70S6K.

 2.

- Total Akt blot has been added to figure 1F
- Total p70S6K has been added to figure 1F
- Blots in figure 1G (=new figure 1K) have been quantitated, except for p110d, which is so weak there is no point to quantify it. Graphs of the quantitation have been added to figure 1 (new figures 1L-P). n=4 in these quantification graphs, i.e we have added more samples for each condition to cover small variations in the immunoprecipitation and western blot experiments.
- p85b has not been added for the same reason as in figure 1E, see above.

3.

- In the quantification graphs added to figure 1F (new figure 1G-J) n=4, ie we have added new experiments with different samples. The WB pictures become very large when adding so many panels, so we decided including all four replicates in the quantification graphs. However, all blots are provided in the raw data sets for the referee to see.
- Same for figure 1G (new figure 1K), i.e n=4. Quantification graphs have been added (new figures 1L-P).

4.   The plates were normalized to a reference sample = active p110a/p85 complex (see original plate pictures in the raw data sets).

 5. Can not find this error. Please specify.
